# Calcium intake from diet and supplement use during early pregnancy: the Expect study I

**DOI:** 10.1007/s00394-019-01896-8

**Published:** 2019-01-19

**Authors:** Jessica P. M. M. Willemse, Linda J. E. Meertens, Hubertina C. J. Scheepers, Nina M. J. Achten, Simone J. Eussen, Martien C. van Dongen, Luc J. M. Smits

**Affiliations:** 1 Department of Epidemiology, CAPHRI Care and Public Health Research Institute, Maastricht University, PO Box 616, 6200 MD Maastricht, The Netherlands; 2 Department of Obstetrics and Gynaecology, Maastricht University Medical Centre, PO 5800, 6202 AZ Maastricht, The Netherlands; 3 Maastricht University, PO 616, 6200 MD Maastricht, The Netherlands; 4 CARIM School for Cardiovascular Diseases, Maastricht University, Maastricht, The Netherlands

**Keywords:** Calcium intake, Pregnancy, Diet, Supplements, Dairy products, Prenatal vitamins

## Abstract

**Purpose:**

Adequate calcium intake during pregnancy is of major importance for the health of both mother and fetus. Up to date, evidence on the prevalence of inadequate calcium intake among pregnant women is sparse for Western countries, and it is unknown to what extent inadequate dietary calcium intake is adequately balanced by supplement use. The objective of this study was to estimate calcium intake from diet and supplement use during the early pregnancy in The Netherlands.

**Methods:**

As part of the Expect cohort study, 2477 pregnant women (8–16 weeks of gestation) completed an online questionnaire including questions on baseline characteristics, the use of calcium containing supplements, and a short food-frequency questionnaire (FFQ). Intake data were used to calculate median calcium intakes from diet, from supplements, and combined, and to compare these values with currently accepted requirement levels.

**Results:**

Forty-two percent of the pregnant women had a total calcium intake below the estimated average requirement of 800 mg/day. Median (interquartile range) calcium intake was 886 (611–1213) mg/day. Calcium or multivitamin supplements were used by 64.8% of the women at 8 weeks of gestation, with a median calcium content of 120.0 (60.0–200.0) mg/day. Prenatal vitamins were the most often used supplements (60.6%).

**Conclusions:**

Forty-two percent of Dutch pregnant women have an inadequate calcium intake. Supplements are frequently used, but most do not contain sufficient amounts to correct this inadequate intake.

## Introduction

Fetal growth places high demands on maternal calcium status [1, 2]. Although part of the demand is met by means of increased intestinal calcium absorption [3–5], adequate calcium intake by the mother remains important. Insufficient calcium intake poses risks to both fetus and mother. Fetal risks include restricted intrauterine growth, low birth weight, poor bone mineralization, and preterm birth, whereas maternal risks include hypertension and preeclampsia [6, 7]. Several trials have shown the beneficial effects of calcium supplementation in the prevention of preeclampsia [8–12].

Recommended calcium intake varies between countries from 900 to 1200 mg/day [13, 14]. The World Health Organization (WHO) and the Food and Agriculture Organization (FAO) of the United Nations recommend a dietary intake of 1200 mg/day of calcium for pregnant women and 1000 mg/day for non-pregnant adults (19–50 years old) [5, 15]. In The Netherlands, the recommended dietary allowance (RDA) is 1000 mg/day for all adults, irrespective of pregnancy status [16].

Diet is the main contributor to total calcium intake [17]. Large differences in calcium intakes between countries have been linked with diversity in food habits and access to food [18]. A WHO survey showed dietary calcium intake to be inadequate (< 1000 mg/day) among 89% of nulliparous pregnant women in developing countries [18]. In view of the good availability and affordability of dairy products in The Netherlands, calcium intake might be expected to be adequate, and likewise in other Western countries. Nevertheless, in a previous Dutch study among pregnant women, mean dietary calcium intake of Dutch pregnant women was found to be just above 1100 mg/day with a standard deviation of 311 [19], indicating that, assuming a normal distribution, one in every six women may have an intake below the estimated average requirement of 800 mg/day [20].

Dietary calcium intake can be complemented with the intake of calcium-containing food supplements. The WHO currently recommends calcium supplementation as part of antenatal care for women with an inadequate dietary calcium intake to lower the risk of developing preeclampsia [5]. To our knowledge, only a few studies analyzed total daily calcium intake from both food and supplement use [21, 22]. Moreover, these studies did not evaluate a large cohort in a Western country. It is also unclear to what extent calcium is advised or prescribed in Dutch clinical practice or how much elementary calcium is ingested from both diet and supplements by Dutch pregnant women. In a small survey, we found that 89% of the gynecologists (*n* = 18) and 10% of the midwives (*n* = 30) counsel their patients on calcium supplements. Most counseling (74%) was directed, toward pregnant women at high risk for preeclampsia.

The main purpose of this study was to estimate total daily calcium intake from both food and supplement use among Dutch women during the early pregnancy. To this end, we used data from a population-based pregnancy cohort. Our study’s second purpose was to evaluate the calcium content of currently used supplements.

## Subjects and methods

### Study population

Data were collected as part of the Expect study I, a prospective cohort study performed in the south-eastern part of the Netherlands with the purpose of validating a number of first-trimester obstetric prediction models. Pregnant women were recruited in 36 midwifery practices and 6 hospitals between July 2013 and January 2015, with follow-up until December 2015. Eligibility criteria for the Expect study were: less than 16 weeks of gestation and a minimum age of 18 years. The Medical Ethical Committee of the Maastricht University Medical Centre evaluated the study protocol and declared that no ethical approval was necessary (MEC 13-4-053). All participating women gave online informed consent.

**Fig. 1:**
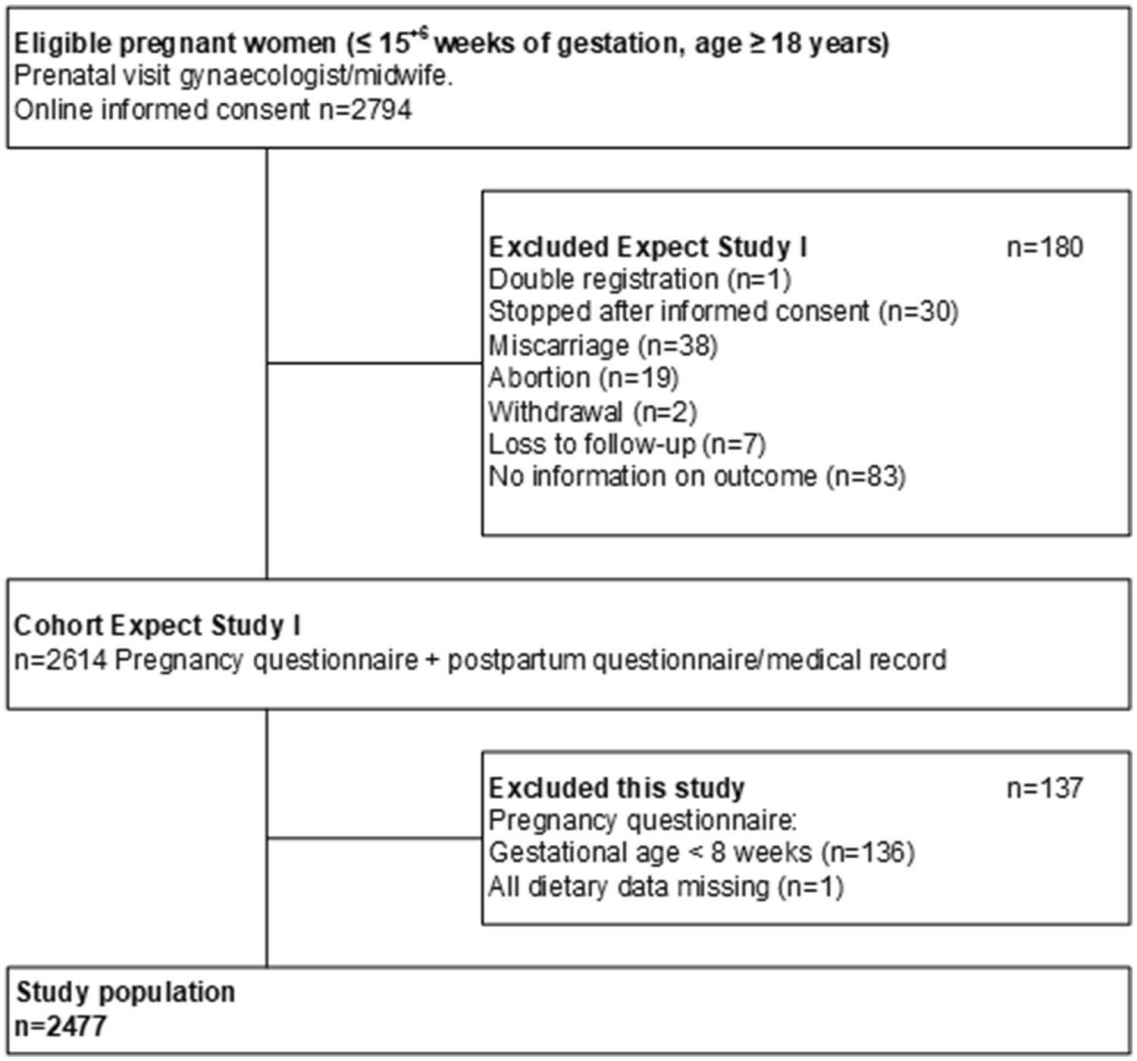
Inclusion flowchart

Figure 1 shows the flowchart of the study population. For this study, we included only women that filled out the questionnaire at 8 weeks of gestation or later, allowing most women to have adapted their diet or started using supplements. On the basis of this restriction, 136 women had to be excluded. We also excluded one woman with the missing values in all dietary questions. Finally, 2477 women were available for analysis.

### Data collection

Women were asked to complete an online questionnaire before 16 weeks of gestation (or a paper version if requested), containing questions about a range of variables including socio-demographic characteristics, lifestyle, obstetric history, medical conditions, and family history. Supplement use before and during pregnancy was asked for, focusing on folic acid, vitamin D, prenatal vitamins, general multivitamins, and calcium preparations, as well as dietary intake of calcium and vitamin D.

### Dietary intake

The Dutch FFQ-TOOL™ (FFQ = food-frequency questionnaire) was used for the selection of food products contributing to calcium and vitamin D intake [21]. For the present study, we only focused on calcium intake. Based on food and nutrient intake data for 20–45 year old (non-pregnant) women who participated in the Dutch National Food Consumption Survey 2007–2010 (DNFCS 2007–2010) and food composition data from the Dutch Food Composition (NEVO) Table 2010, which were embedded in the Dutch FFQ-TOOL™, we made the FFQ-TOOL select those food products that cumulatively covered > 80% of the variance in calcium intake [23]. The selection procedure resulted in 18 food items, for which both the frequency of use (reference period: last month) and the average daily amount of use were asked: milk and buttermilk; yoghurt and fromage frais (with or without fruit); yoghurt drinks and other dairy beverages; chocolate milk; custard and pudding; Dutch cheese; non-Dutch cheese and cream cheese; cheese spread; bread spread (sub types: margarine; low-fat margarine; and butter); cooking fat (bake and fry products); and fish (subtypes: fat fish such as salmon, mackerel, eel, and white harring; lean fish such as codfish, tilapia, panga fish and trout; white fish filet; smoked or steamed fish; herring; and fish fingers. Milk, milk products, and cheese were included in the questionnaire as major sources of calcium, bread spread, bake and fry products, and fish as major sources of vitamin D.

We chose to include questions on food products that cumulatively cover > 80% of the variance in calcium intake and eliminate food products with small calcium contents. This consideration was made to encourage participants to complete the full questionnaire of the Expect study I. Covering the complete dietary calcium intake in this study was not feasible, as the FFQ was part of an intensive questionnaire containing several pregnancy-related topics, with the purpose of validating a number of first-trimester obstetric prediction models. The food items in the questionnaire covered an estimated 62% of total absolute dietary calcium intake. In case dietary calcium intakes were either 0 mg/day or over 1750 mg/day, women were contacted to check whether any unintended errors were made. In the group of women with dietary intakes above the tolerable upper level of 2500 mg per day, four women reported correct intakes and the rest made an unintended error, which was corrected after contact with the participant. Since intakes < 200 mg calcium in the mostly Caucasian population of this study are relatively low, we checked whether exclusion of these outliers (dietary calcium intake < 200 mg, *n* = 106) affected median dietary calcium intake and the percentage of total inadequate intake in an extra-sensitivity analysis.

### Supplements

Questions on potential calcium-containing supplements such as prenatal vitamins, general multivitamins, and calcium supplements, were included for this study. We requested time and period of use (start of usage before and during pregnancy, when potentially stopped, current use), brand and any subtype, frequency of use per week, and amount of tablets per day.

Calcium was standardized to the elemental form in milligrams, based on the labels. We contacted the manufacturers for the clarification when the exact elementary amount of calcium in the supplement was unclear.

### Data analysis

Baseline characteristics were analyzed and presented as percentages. Missing values in the baseline characteristics of the Expect I cohort regarding education level (*n* = 3) and body mass index (BMI) (*n* = 5), were imputed using stochastic regression imputation based on predictive mean matching [24].

We calculated individual daily dietary calcium intake by multiplying frequency of consumption by consumed amounts of all the assessed food products and combining product intake (grams per day) with calcium content of each product according to the Dutch Food Composition Table of 2010 (NEVO-online 2010) [25] and DNFCS2007-2010 [23]. Missing frequency and amount values were imputed with the modal value of all the valid values for the specific variable. To account for the incomplete coverage of the food-frequency questionnaire, we adjusted the estimated calcium intake values (estimated intake*100/61.65). In this way, adjusted total calcium intakes were used in the analyses and presented in the results.

We calculated percentages of women using the different calcium-containing supplements for each week of gestation. We calculated daily calcium intake from supplement use by combining frequency, amount of supplements, and content of specific supplements among current users at 8 weeks of gestation. In case a participant used a supplement but did not know the exact (subtype) brand, the modal value was imputed. Median values of calcium supplement intake at 8 weeks of gestation were calculated.

Median values of total calcium intake, dietary calcium intake, and calcium intake from supplement use were calculated and presented in milligrams per day, with interquartile range (IQR). Total calcium intake was compared to the Recommended Dietary Allowance (RDA) of 1000 mg/day and the Estimated Average Requirement (EAR) of 800 mg/day. An intake level of 800 mg calcium is expected to satisfy the needs of 50% of all pregnant women [26, 27]. The EAR cut point method proposed by the Institute of Medicine (IOM) was used to assess the level of inadequacy in calcium intake in our population [20, 28].

Analyses were performed using IBM SPSS statistics version 23.

## Results

### Study population

The baseline characteristics of the study population (*n* = 2477) are shown in Table 1. Almost all women were of Caucasian origin, more than 80% of the women were aged 26–35 years, and 55% had a finished tertiary level of education. Half of the study population was nulliparous and 54% of the women had a normal prepregnancy BMI (20.0–24.9).

**Table 1:** Baseline characteristics of the study population

Characteristics	*N* = 2477^a^ (%)
Age (years)	
18–25	11
26–30	43
31–35	38
> 35	9
BMI before pregnancy	
< 20.0	13
20.0–24.9	54
25.0–29.9	22
> 30.0	11
Ethnicity	
Caucasian	97
Asian	0.8
Negroid	0.1
Hispanic	0.4
Mixed	2
Parity	
Nulliparity	51
Level of education	
Tertiary	55

^a^Percentages do not always add up to 100% due to rounding

### Total calcium intake

**Fig. 2:**
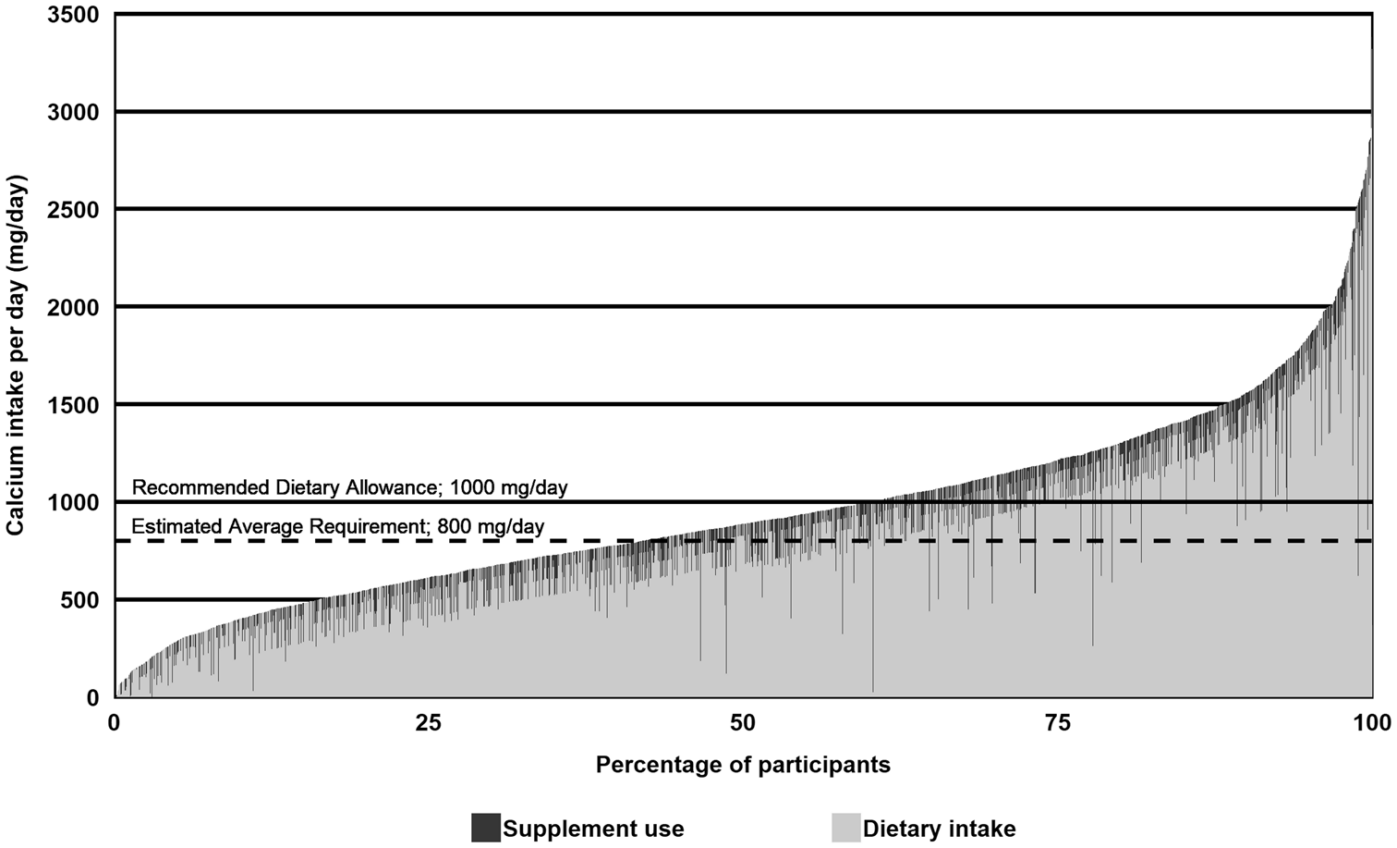
Calcium intake from diet and supplement use at 8 weeks of gestation

Median (IQR) calcium intake was 886 (611–1213) mg/day. Forty-two percent of the women (*n* = 1045) had a total calcium intake below the EAR and 60% (*n* = 1489) did not meet the RDA of 1000 mg calcium per day (Fig. 2).

### Dietary calcium intake

Diet was the main contributor to total calcium intake (Fig. 2). Almost all women (99.8%) used calcium-containing dietary products. Dietary calcium intake varied from a minimum of 0 mg to a maximum of 4215.8 mg calcium per day. Median (IQR) dietary calcium intake was 798 mg/day (530–1113). Most contributing to dietary calcium intake were consumption of Dutch cheese (31%) and milk (27%).

Based on just food intake, 50% of the women had an intake below the EAR of 800 mg/day. In this group, 16% added enough calcium from supplements to their diet to reach the EAR.

### Supplement use

**Fig. 3:**
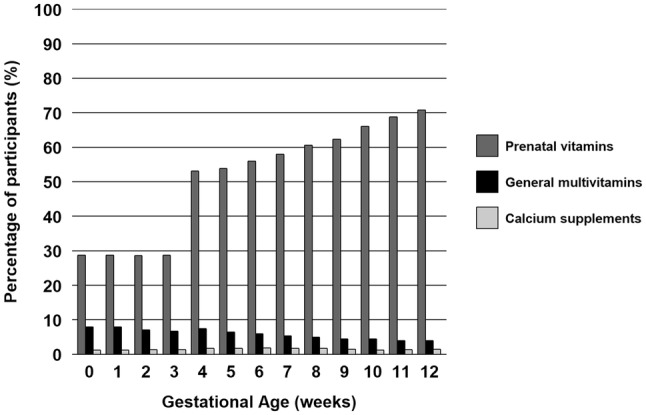
Calcium-containing supplement use per gestational age

Supplement use per category varied before and during early pregnancy (Fig. 3). Before pregnancy, 29% of the women started the use of prenatal vitamins, and at 8 weeks of gestation, this type of supplement was used by 61%. On the other hand, the use of general multivitamins declined from 8% usage before pregnancy to 5% at 8 weeks of gestation. The use of specific calcium supplements did not show much variation. Supplements containing only calcium were used by only 1% of the women before pregnancy and 2% used them at 8 weeks of gestation.

### Calcium content of supplements

Median (IQR) calcium intake from supplement use at 8 weeks of gestation was 120 mg/ day among users (60–200).

The median (IQR) calcium intake from prenatal vitamins at 8 weeks of gestation (61%) was 120 mg/day (60–200). The calcium content of various calcium-containing prenatal vitamins ranged from 60 to 326 mg calcium per tablet. Three of twenty-three prenatal vitamins did not contain calcium.

For general multivitamins, the median (IQR) calcium intake was 103 mg/day (5–162) at 8 weeks of gestation. The content of the 66 reported general multivitamins ranged from 10 to 500 mg per tablet and 18 reported general multivitamins did not contain any calcium.

The median (IQR) calcium intake from calcium supplements at 8 weeks of gestation (2%) was 337 mg/day (214–508). The content of reported calcium supplements ranged from 240 to 1000 mg calcium per tablet.

### Sensitivity analysis

Exclusion of participants with dietary calcium intakes < 200 mg (*n* = 106) resulted in a median (IQR) dietary calcium intake of 824 (570–1129) mg and a median (IQR) total calcium intake of 906 (649–1229) mg per day. The percentage of women with a total calcium intake below the EAR was 40%, differing not much from the percentage in the total population.

## Discussion

### Main finding

We found that 42% of the population had a total calcium intake below the EAR of 800 mg calcium per day.

### Previous findings on calcium intake

The beneficial effects of an adequate calcium intake during pregnancy have been a topic of interest for quite a long time [6, 7, 29–35]. Dietary calcium intake during pregnancy has been mainly assessed among populations in the developing countries, and lowest intakes were predominantly found in Asia and Africa [18]. A WHO systematic review from 2005 showed that the average dietary calcium intake in developed countries was below the RDA in half of the included studies [18]. More recent studies showed even higher proportions of inadequate calcium intake in women of reproductive age in the developed countries with inadequacy in more than half of the population [31, 32].

Two previous studies have looked at calcium intake among women in The Netherlands. Among pregnant women from Rotterdam, mean dietary calcium level in the first trimester of pregnancy was found to be greater than results of our current study show, namely 1108 (sd, 311) [18]. Assessment of dietary calcium intakes in this study was done more extensively, by means of a modified FFQ containing 293 food items. On the assumption of a normal distribution of calcium intakes, these figures would indicate that about one in every six women would have an intake below EAR, which is a lower proportion than found in our study. However, as observed in our study, intakes may be strongly right-skewed, implying a higher proportion of women with sub-EAR calcium intakes. Furthermore, the women included in the Rotterdam study had a more diverse ethnicity and data on supplement use are lacking.

In the Dutch National Food Consumption Survey 2007–2010, more than one-fourth of the women of reproductive age (age categories 19–30 and 31–50 years) did not reach the EAR of 800 mg calcium per day by diet alone. Calcium-containing supplements were used by 4–8% [23]. These women were, however, from the general (non-pregnant) population, while pregnancy may cause nausea and changes in food choices [36], possibly explaining the lower intake in our population. Next to this, while we cover 62% of total absolute dietary calcium intake with our inquired food items, the Food Consumption Survey included a more precise method, using two non-consecutive 24-h dietary recalls. The differences in population and methods may have contributed to the < 17% difference in the amount of women with inadequate intake.

Even though the used assessments for dietary calcium intake in the previously mentioned studies were more detailed and perhaps more accurate, our study points into the important direction of an inadequate calcium intake in a Western population. In addition, to our knowledge, the current study is the first one that assessed the complete calcium intake from both diet and supplement use in Dutch pregnant women.

### Implications

We showed that dietary sources alone are insufficient to meet an adequate calcium intake in 50% of the pregnant women. Ingestion of the amount of calcium necessary to reach the EAR is not easily achieved via diet alone [31]. Although dairy products are the largest contributor to calcium intake, 100 g milk contains only 108–200 mg calcium [25]. Changing dietary habits is challenging for many women despite a presumed higher awareness of health issues during pregnancy [37, 38].

Dietary intake can be complemented by supplements to achieve an adequate calcium intake. Supplement use will result in a higher prevalence of an adequate calcium intake [32, 39]. In the current study, 74% of our participants used multivitamin or calcium supplements. Morisset et al. [40] demonstrated calcium-containing supplements are being used by 73% of their study population (*n* = 1186), which is comparable to our study population. However, the calcium content of the most frequently used supplements—prenatal vitamins—is insufficient to complement the dietary calcium intake to a level that meets EAR. Remarkably, 3 of the 23 used prenatal vitamins did not contain any calcium. Moreover, the effectiveness of prenatal vitamins other than separate folic acid and vitamin D supplements has never been proven [41]. Because of the insufficient calcium content of most prenatal vitamins and multivitamins, it seems essential to advise the use of separate calcium supplements for pregnant women with an inadequate calcium intake. We recently showed that advising all pregnant women to use calcium supplements can be expected to cause substantial reductions in the incidence of preeclampsia as well as related health care costs [42]. No major side effects have been described, but the tolerable upper level of 2500 mg calcium per day should be taken into account, since hypercalcemia could cause renal insufficiency, vascular and soft-tissue calcification, hypercalciuria, and kidney stones [20, 23].

### Strengths and limitations

Our study provides an overview of calcium intake levels by pregnant women based on not just diet but also supplement use. A major strength of our study is the large sample size of 2477 participants. In our population, women of Caucasian origin were somewhat overrepresented and more than half of the population had a high educational level, which corresponds to the composition of the population of Dutch women in their thirties [43]. A previous Canadian study showed that calcium intake was lower in women with lowest educational levels [40], so the overrepresentation of high educated women in our study may have led to an underestimation of the total percentage of women with an inadequate intake.

A few limitations of our study should be addressed. First, since there is no biochemical assay to display the nutritional calcium status, we had to depend on questionnaires. Repeated dietary recalls or records might have been considered as more accurate approaches for food intake assessment. However, this method would not be achievable in a large cohort. Nevertheless, the FFQ method is widely used for food product and nutrient intake assessment, and although its main strength is in the ranking of individuals according to their intakes of frequently used foods and nutrients, it is also considered a feasible tool to gain insight in the percentage of inadequate intake in a large population [44]. Second, our assessment of dietary calcium intake included only those products with the highest contribution to their calcium content to minimize the load of the questionnaire. Moreover, the selected products inquired in our questionnaire contributed to more than 60% of all dietary calcium intake and we recalculated total calcium intake to 100% [45]. This selection procedure probably has resulted in an underestimation of dietary calcium intake, requiring adjustment [40]. The five most contributing food products to dietary calcium intake that were not inquired in our study were bread (3.9%), water (3.7%), cooked or stir fried vegetables (3.5%), coffee (2.2%), and tea (1.6%). Even though the contribution of these food products to total calcium intake is limited and already covered in the recalculation from 61.65 to 100%, we compared our methods to the methods used in a more extensive FFQ. To ensure that our methods estimated the correct total calcium amount, we applied our methods to the data of pregnant women from an older birth cohort study (*N* = 2855) [46]. Thirty-nine from the 213 food items from the KOALA-FFQ were comparable to the food items which we inquired in our study. Total calcium intake based on the 39 food items was strongly correlated to calcium intake based on the complete KOALA-FFQ (Pearson’s *r* = 0.95). Women who did not consume food products inquired in our questionnaire were contacted and have a recalculated total dietary calcium intake of 0 mg, while they may have consumed other calcium-containing products. However, sensitivity analysis did not show large differences after exclusion of outliers. Third, recall may not have been optimal as dietary intake was inquired for the month prior to filling in the questionnaire. Finally, there may have been intra-individual variation in food intake which was not covered by the applied measurement procedure. Food intake may vary over time and, perhaps, especially during pregnancy as women may experience sickness in the early pregnancy. However, the previous evidence showed that calcium intake from supplement use does not differ much across trimesters [47].

## Conclusion

Our study provides insight in calcium intake from both diet and supplement use during the early pregnancy. We found that 42% of the pregnant population had an inadequate total calcium intake. Efforts to reach an adequate calcium intake are recommendable for all women with inadequate calcium intakes. We advise further research into barriers and facilitators for obtaining an adequate calcium intake during pregnancy. In addition, research into risk indicators for an inadequate calcium intake could be useful to implement directed health promotion to target groups.
